# Evaluation of Laboratory and Sonographic Parameters for Detection of Portal Hypertension in Patients with Common Variable Immunodeficiency

**DOI:** 10.1007/s10875-022-01319-0

**Published:** 2022-07-11

**Authors:** Anna-Maria Globig, Valentina Strohmeier, Rambabu Surabattula, Diana J. Leeming, Morten A. Karsdal, Maximilian Heeg, Gerhard Kindle, Sigune Goldacker, Caroline von Spee-Mayer, Michele Proietti, Birke Bausch, Dominik Bettinger, Michael Schultheiß, Robert Thimme, Detlef Schuppan, Klaus Warnatz

**Affiliations:** 1 Department of Medicine II, Gastroenterology, Hepatology, Endocrinology, and Infectious Diseases, University Medical Center Freiburg, Faculty of Medicine, University of Freiburg, Freiburg, Germany; 2 Department of Rheumatology and Clinical Immunology, Medical Center, Faculty of Medicine, University of Freiburg, University of Freiburg, Breisacher Str. 115, 79106 Freiburg, Germany; 3 Center for Chronic Immunodeficiency (CCI), Medical Center, Faculty of Medicine, University of Freiburg, University of Freiburg, Freiburg, Germany; 4 Faculty of Biology, University of Freiburg, Schaenzlestrasse 1, Freiburg, Germany; 5 Institute of Translational Immunology and Research Center for Immune Therapy, Mainz University Medical Center, 55131 Mainz, Germany; 6 Nordic Bioscience Biomarkers and Research, Herlev, Denmark; 7 Institute for Immunodeficiency, Medical Center - University of Freiburg, Faculty of Medicine, University of Freiburg, Freiburg, Germany; 8 Department of Rheumatology and Immunology, Hannover Medical School, Hannover, Germany; 9 Cluster of Excellence RESIST (EXC 2155), Hannover Medical School, Hannover, Germany; 10 Division of Gastroenterology, Beth Israel Deaconess Medical Center, Harvard Medical School, Boston, MA 02215 USA

**Keywords:** Common variable immunodeficiency, hepatopathy, portal hypertension, diagnosis, nodular regenerative hyperplasia

## Abstract

Timely detection of portal hypertension as a manifestation in a subgroup of patients with common variable immunodeficiency (CVID) represents a challenge since it is usually not associated with liver cirrhosis. To identify relevant markers for portal hypertension, we evaluated clinical history, laboratory parameters, and abdominal ultrasound including liver elastography and biomarkers of extracellular matrix formation. Twenty seven (6%) of 479 CVID patients presented with clinically significant portal hypertension as defined by either the presence of esophageal varices or ascites. This manifestation occurred late during the course of the disease (11.8 years after first diagnosis of CVID) and was typically part of a multiorgan disease and associated with a high mortality (11/27 patients died during follow up). The strongest association with portal hypertension was found for splenomegaly with a longitudinal diameter of > 16 cm. Similarly, most patients presented with a liver stiffness measurement (LSM) of above 6.5 kPa, and a LSM above 20 kPa was always indicative of manifest portal hypertension. Additionally, many laboratory parameters including Pro-C4 were significantly altered in patients with portal hypertension without clearly increasing the discriminatory power to detect non-cirrhotic portal hypertension in CVID. Our data suggest that a spleen size above 16 cm and an elevated liver stiffness above 6.5 kPa should prompt further evaluation of portal hypertension and its sequelae, but earlier and better liquid biomarkers of this serious secondary complication in CVID are needed.

**Supplementary Information:**

The online version contains supplementary material available at 10.1007/s10875-022-01319-0.

## Introduction

Common variable immunodeficiency (CVID) is the most common form of symptomatic primary immunodeficiencies [1]. CVID is a genetically and immunologically heterogeneous disease that is defined by hypogammaglobulinemia of IgG and IgA with or without low IgM levels [2]. Patients can be classified according to molecular characteristics, immunologic phenotype, or clinical characteristics [3–10]. Based on clinical phenotype, patients can be divided into two subgroups: Those that show primarily infectious complications and those with additional disease manifestations such as enteropathy, liver disease, interstitial lung disease, granuloma, splenomegaly, autoimmunity, or malignancy. It is essential to distinguish these patient subgroups as patients with additional non-infectious complications have a significantly decreased life expectancy [11, 12].

While infectious complications, lung function, cytopenias, gastrointestinal involvement, and immunoglobulin levels are more closely monitored by clinicians, affection of the liver with development of portal hypertension is a severe complication that is usually recognized only at advanced stages. Importantly, diagnostic markers and therapeutic approaches in fibrotic liver disease and portal hypertension due to CVID are insufficiently defined [13]. Chronic liver disease has been reported to occur in ~ 12% of CVID patients seen in tertiary treatment centers, and occurrence of liver disease is associated with increased mortality [11, 12, 14]. Underlying pathologies comprise granulomatous inflammation, autoimmune hepatitis, and most commonly nodular regenerative hyperplasia (NRH) [15]. While liver function may be still preserved, the major complication of NRH is portal hypertension with development of ascites and/or esophageal varices that can ultimately cause potentially fatal upper gastrointestinal bleeding [13, 16, 17].

With regard to laboratory markers of liver disease in CVID, especially elevations in alkaline phosphatase (ALP), bilirubin and transaminases have been described [18, 19]. Abdominal ultrasound and magnetic resonance imaging as well as transient elastography (FibroScan®) can be used to assess structural changes in the liver as well as signs of portal hypertension [20–22]. There is, however, so far no standard in the field with regard to which parameters should be assessed in CVID patients to determine whether the patient suffers from portal hypertension. In this study, we therefore characterized patients with CVID who developed portal hypertension in a cohort of 479 CVID patients treated at the University Hospital of Freiburg, Germany, and longitudinally assessed conventional and novel laboratory, clinical, endoscopic, and ultrasound characteristics of these patients.

## Methods

### Patients

Patients with CVID were retrospectively identified from the hospital information system of the University Hospital of Freiburg according to documentation from the outpatient department of the Centre for Chronic Immunodeficiency. Laboratory data and clinical data as well as ultrasound and endoscopy data for these patients were exported and manually validated from the hospital information system. Clinically significant portal hypertension was defined as occurrence of esophageal varices and/or portal hypertensive gastropathy and/or ascites. Female patients with a single episode of ascites in the recto-uterine pouch due to gynecological reasons were not included in the patient group with portal hypertension. The date of onset of portal hypertension was identified according to the date of the first documentation of the aforementioned symptoms. If documentation from previous inpatient or outpatient stays in other hospitals was available, the date of onset was corrected to the first description of aforementioned signs. Patients that never showed esophageal varices, portal hypertensive gastropathy, or ascites were assigned to the control group.

In total, we identified 479 CVID patients of whom 27 showed clinical signs of portal hypertension (for details, see Table 1). Not all data were available for every patient; the number of available data points is indicated in the figures.

**Table 1:** Clinical characteristics of CVID patients with portal hypertension

Patient	Gender	Year of birth	Manifestation of CVID	Diagnosis of CVID	Genetics	Euroclass	Freiburg class	Onset portal hypertension	Liver biopsy	Etiology of liver disease (pathology report)	Maximal LSM (kPa)	Death
1	m	1968		1996	idiopathic	B + 21norm smB-	Ib	2004	Yes	Autoimmune cholangitis	n.a	Yes
2	m	1963		1991	n.a	B + 21norm smB-	Ib	2015	Yes	Discrete portal inflammatory reaction	n.a	Yes
3	m	1956	1982	1996	NFKB1 deficiency	B + 21low smB +	Ia	1999	Yes	HCV associated liver cirrhosis	30.8	No
4	f	1969	1972	2003	n.a	B + 21low smB +	Ia	2004	Yes	Nodular regenerative hyperplasia	16.8	Yes
5	f	1959	1998	2001	n.a	B + 21low smB-	Ia	2011	Yes	Nodular regenerative hyperplasia	n.a	Yes
6	f	1948	1986	1996	n.a	B + 21low smB-	Ia	2004	Yes	Nodular regenerative hyperplasia	n.a	Yes
7	f	1956	1996	2006	n.a	nd	Ib	2008	Yes	Chronic portal and lobular hepatitis	26.3	Yes
8	f	1966	1990	1998	n.a	B + 21low smB-	Ib	2012	Yes	Nodular regenerative hyperplasia	n.a	Yes
9	m	1956	1996	2003	n.a	B + 21low smB-		2012	Yes	Discrete hepatitis with periportal and discrete intralobular inflammatory infiltrates	9.9	Yes
10	m	1971	2004	2006	n.a	B + 21low smB-	Ia	2015	Yes	Granulomatous liver disease with epitheloid granuloma and lymphocytic portal-inflammatory infiltrate	22.3	No
11	f	1961	1999	2002	n.a	B + 21low smB-	Ia	2012	Yes	Nodular regenerative hyperplasia and suspected autoimmune cholangiopathy	46.4	No
12	m	1962		1977	ICOS deletion	B + 21norm smB-	Ib	2015	Yes	Nodular regenerative hyperplasia	9.9	No
13	m	1956	1984	1991	NFKB1 deficiency	B + smB-	Ia	2017	Yes	Nodular regenerative hyperplasia	7.3	Yes
14	f	1959	2000	2005	n.a	B + 21low smB-	Ia	2017	No	n.a	8.9	No
15	m	1955	2007	2012	n.a	B + 21low smB-	Ia	2017	Yes	Nodular regenerative hyperplasia	11.5	No
16	m	1978	2010	2012	idiopathic	B + 21low smB-	Ia	2018	Yes	Nodular regenerative hyperplasia	n.a	No
17	f	1972		1995	n.a	B-		2015	Yes	Nodular regenerative hyperplasia	n.a	No
18	m	1968		1989	n.a	nd	Ia	2004	Yes	CVID involvement of liver	n.a	Yes
19	f	1960	1988	1993	TACI deficiency	B + 21low smB-		2009	Yes	Cryptogenic liver cirrhosis	8.7	No
20	f	1976		1986	n.a	B + 21low smB-	Ia	2010	Yes	Nodular regenerative hyperplasia	7.4	No
21	m	1962		2004	idiopathic	nd		2010	No	n.a	n.a	No
22	f	1983		2000	n.a	B + 21low smB-	Ia	2011	No	n.a	n.a	No
23	f	1957	1962	1996	n.a	B + 21low smB-	Ia	2008	Yes	Granulomatous liver disease with epitheloid cell granuloma	23.6	Yes
24	m	1993		2011	idiopathic	B + 21low smB-	Ia	2016	No	n.a	n.a	No
25	f	1993	2006	2014	CTLA-4 deficiency			2017	No		15.7	No
26	m	1963		2013	n.a	B + 21low smB-		2017	No	n.a	n.a	No
27	m	1983		2014	idiopathic	B + 21norm smB-		2018	Yes	Granulomatous hepatitis	30.7	No

*n.a.*, not available; *NFKB1*, nuclear factor kappa B subunit 1; *ICOS*, inducible T cell costimulator; *TACI*, transmembrane activator and calcium-modulator and cyclophilin ligand interactor; *CTLA-4*, cytotoxic T-lymphocyte associated protein 4; *HCV*, hepatitis C virus.

### Transient Elastography

Where available, results of transient elastography (FibroScan®) measurements were included in the study. This ultrasound-based technique using a hand-held ultrasound probe allows non-invasive measurement of liver stiffness. Interpretation of generated data depends on the underlying disease-pathology [23–27]; however, transient elastography has previously been demonstrated to be of diagnostic value for CVID patients, particularly in those at risk for portal hypertension [20, 28]. ROC analysis of the LSM was performed, and the optimal cutoff for determination of portal hypertension was calculated by the Youden Index.

### ELISAs

Biomarkers of extracellular matrix formation were assessed in serum with validated competitive ELISAs developed and performed as described in previous publications for type III collagen formation (PRO-C3) [29], type IV collagen formation (PRO-C4) [30], and type VI collagen formation and endotrophin (PRO-C6) by Nordic Bioscience (Herlev, Denmark). All samples were measured in duplicates. The median time duration between diagnosis of portal hypertension in the “portal hypertension” group and obtaining the sample was 1051 days, 95% CI [83; 2563].

### Data Analysis

Pseudonymized data were stored in MariaDB 10.4.13. Data analysis was performed using R (version 3.6.1) and the following packages: DBI 1.1.0, dplyr 0.8.3, ggplot2 3.3.0, purrr 0.3.3, tidyr 1.0.0, ggbeeswarm 0.6.0, patchwork 1.0.0, lme4 1.1–21, plotROC 2.2.1, and wesanderson 0.3.6. Statistical tests used are indicated in the figure legends. A *p* value < 0.05 was considered significant. **** indicates a *p* value < 0.0001, *** < 0.001, ** < 0.01, and * < 0.05. For laboratory and continuous ultrasound parameters, a linear mixed model was calculated: lmer(value ~ group + year(date) + (1|PatientID).

## Results

**Fig. 1:**
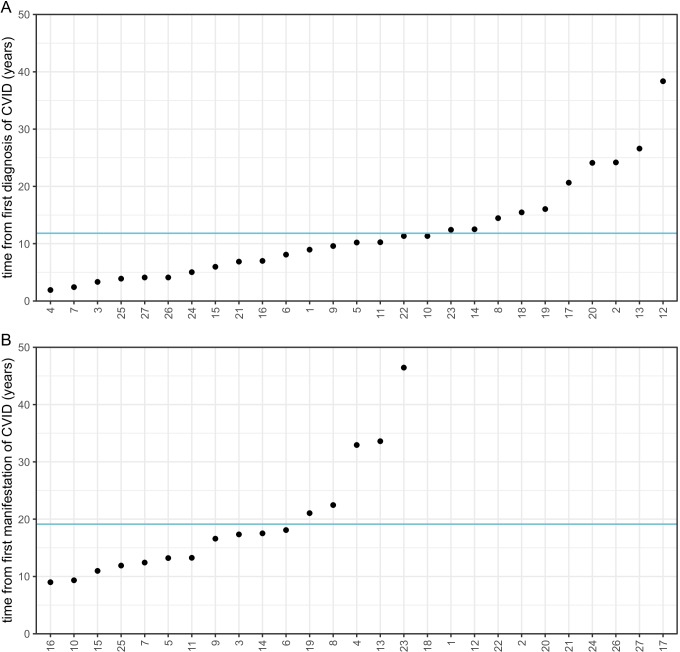
Time from onset of CVID diagnosis and manifestation. Time period between first diagnosis (**A**) and first manifestation (**B**) of CVID and diagnosis of portal hypertension is depicted. Data on date of manifestation of CVID were not available for all patients. The blue line indicates the mean

**Fig. 2:**
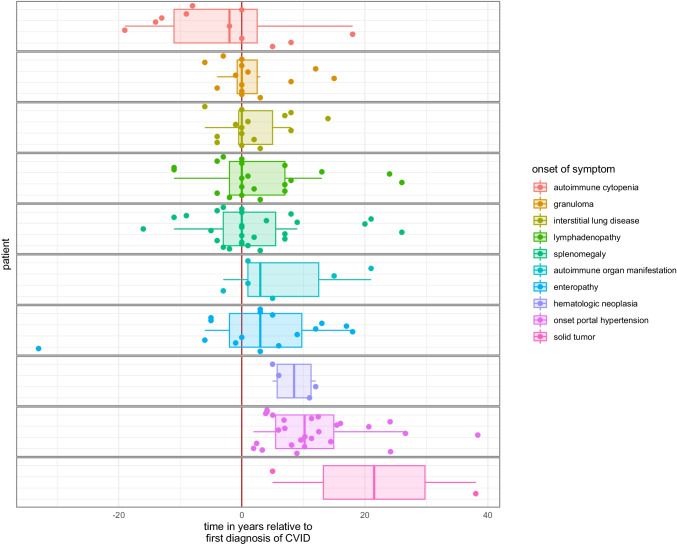
Time of onset of CVID manifestations relative to date of CVID diagnosis. Time of onset of CVID manifestations in patients with portal hypertension was plotted relative to first diagnosis of CVID

In a retrospective analysis of patients treated at the Centre for Chronic Immunodeficiency of the University Hospital of Freiburg, we have identified 479 CVID patients of whom 27 (6%, 13 female and 14 male) showed clinical signs of portal hypertension (see Table 1). Ninety two percent (23 of 25 patients with available data for ascites) of the CVID patients with portal hypertension presented with ascites and 62% (16 of 26 patients with available data for esophageal varicosis) with esophageal varices during the disease course. Liver biopsy was performed in 21 patients, revealing nodular regenerative hyperplasia of the liver in 52% (11/21) and granulomatous liver disease in 14% (3/21) of patients with biopsy. Typical cirrhosis was histologically seen in only 2 patients. On average, signs of portal hypertension first occurred 11.8 years after first diagnosis of CVID and 19.1 years after first manifestation of CVID respectively (Fig. 1). Compared to other manifestations of CVID, signs of portal hypertension appeared markedly later (Fig. 2).

**Fig. 3:**
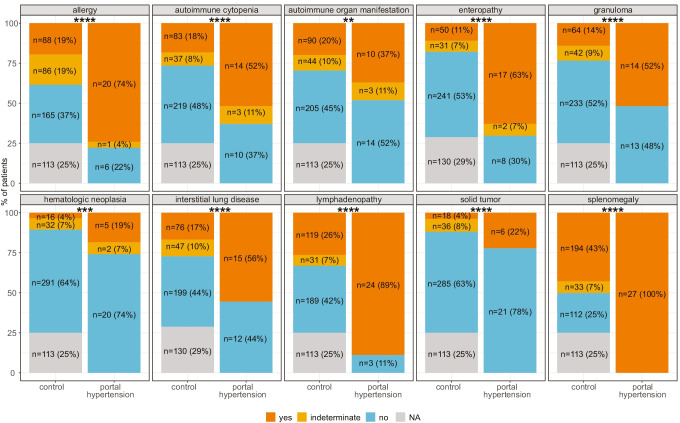
Clinical manifestations of CVID patients with and without portal hypertension. Clinical manifestations of CVID were compared between patients with and without portal hypertension. Fisher’s exact test was used to assess statistical significance

To define which patients are at risk for portal hypertension, we assessed other CVID manifestations in patients with and without portal hypertension. Interestingly, patients with portal hypertension displayed a higher rate of other CVID manifestations such as allergy, autoimmune cytopenia, other autoimmune organ manifestations, enteropathy, granuloma, interstitial lung disease, lymphadenopathy, solid tumors, and splenomegaly compared to patients without portal hypertension (Fig. 3). These findings underline that patients with portal hypertension belong to a cohort with a high risk for multiorgan disease within the total CVID population. Compatible with this association, most of the patients with portal hypertension belonged to the EUROClass group of B + smB-21low as this had been associated before with more complex disease in CVID [6]. The severity of the disease associated with the manifestation of portal hypertension was underlined by the high mortality rate of 41% (11/27) during follow up in this cohort.

**Fig. 4:**
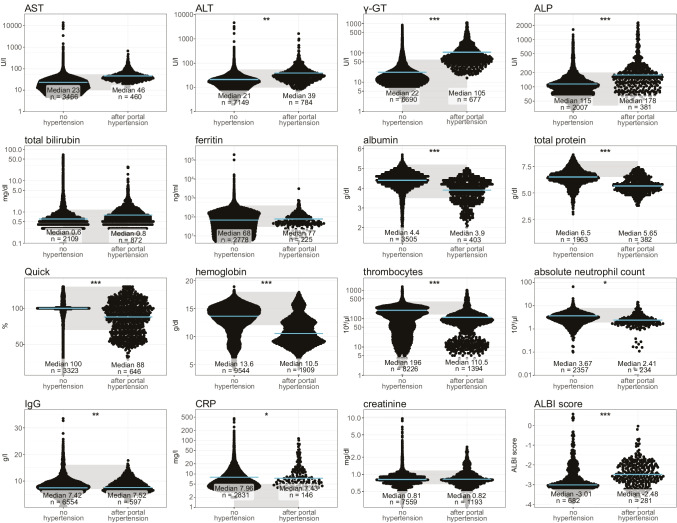
Laboratory parameters of CVID patients with and without portal hypertension. Laboratory values were compared between patients without portal hypertension combined with values of patients before the onset of portal hypertension against values after diagnosis of portal hypertension. A linear mixed model was computed to assess statistical significance. Blue line indicates the median. Grey shaded area reflects normal range

To allow for easier clinical identification of these patients, we next sought to characterize the changes in routine blood-based parameters. Patients after diagnosis of portal hypertension displayed significant increases in alanine transaminase (ALT), gamma-glutamyltransferase (γ-GT), and ALP, while serum albumin and total protein were decreased. Patients after diagnosis of portal hypertension further displayed impaired coagulation (Quick value), lower hemoglobin, and reduced thrombocyte counts. The absolute neutrophil count as well as the CRP levels were reduced after diagnosis of portal hypertension. The ALBI score, a score to assess liver dysfunction calculated based on albumin and total bilirubin [31], was increased in CVID patients after diagnosis of portal hypertension (Fig. 4). However, despite these changes, a notable proportion of measurements was within the normal range, thus complicating the identification of this patient collective.

**Fig. 5:**
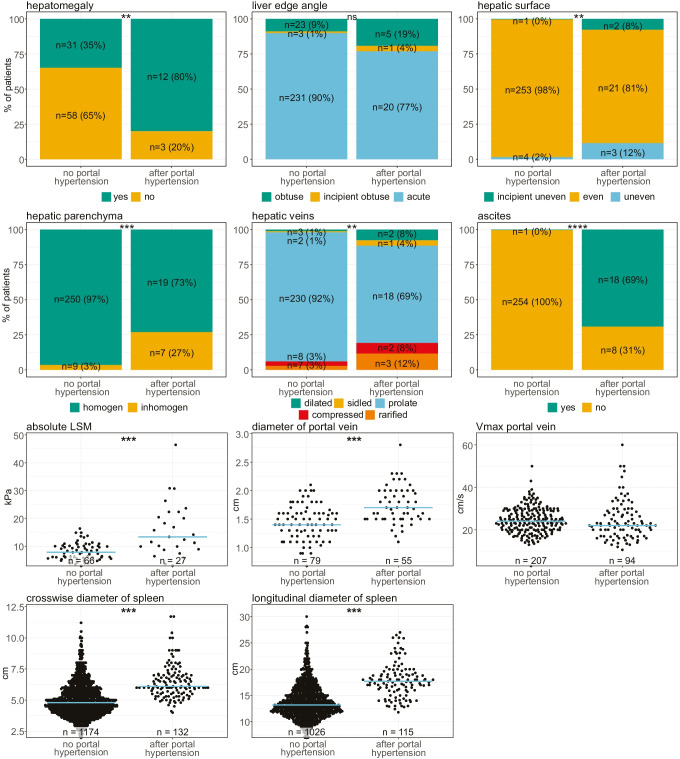
Abdominal ultrasound of CVID patients with and without portal hypertension. Abdominal ultrasound results of patients with and without portal hypertension were compared. For categorial variables, the last available ultrasound of patients without portal hypertension was compared with the first available ultrasound after diagnosis of portal hypertension of patients with portal hypertension. For continuous variables, values were compared between patients without portal hypertension combined with values of patients before the onset of portal hypertension against values after diagnosis of portal hypertension. A linear mixed model was computed to assess statistical significance

**Fig. 6:**
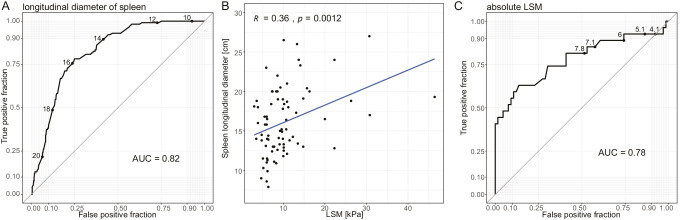
Longitudinal diameter of the spleen as marker for portal hypertension. (**A**) ROC analysis of the longitudinal diameter of the spleen was performed using the values from patients without portal hypertension and from patients after onset of portal hypertension. The optimal cutoff was calculated by the Youden Index (15.7 cm). (**B**) Correlation of the longitudinal diameter of the spleen as determined by ultrasound with the LSM value as determined by FibroScan®. Statistical significance was assessed with Pearson correlation. (**C**) ROC analysis of the absolute LSM was performed using the values from patients without portal hypertension and from patients after onset of portal hypertension. The optimal cutoff was calculated by the Youden Index (11.2 kPa)

In abdominal ultrasound, patients with portal hypertension frequently showed hepatomegaly, while alterations of the hepatic surface and hepatic veins as well as inhomogeneous hepatic parenchyma occurred infrequently. Ascites as one of the defining features of portal hypertension was detected in 92% of the affected patients. Interestingly, the portal vein diameter was increased in patients with portal hypertension but the Vmax of the portal vein was not altered, which is in contrast to other liver disease entities with portal hypertension. Both crosswise and longitudinal diameter of the spleen were significantly increased in patients with portal hypertension (Fig. 5). Since splenomegaly is a common feature of CVID, we were interested to assess whether it can still constitute a marker for portal hypertension in this particular patient collective. ROC analysis of the longitudinal diameter of the spleen resulted in an optimal cutoff for determination of portal hypertension of 15.7 cm and an AUC of 0.82 (Fig. 6A). Additionally, the longitudinal diameter of the spleen correlated significantly with the LSM (liver stiffness measurement, FibroScan®) value of the respective patient (*R* = 0.36, *p* = 0.0012; Fig. 6B).

Transient elastography (FibroScan®) revealed significantly higher liver stiffness measurement (LSM) in CVID patients with portal hypertension when compared to the control cohort. All 15 patients with portal hypertension and elastography had pathological LSM values > 6.5 kPa and LSM > 20 kPa and were only seen in patients with portal hypertension. ROC analysis of the LSM was performed, and the optimal cutoff for determination of portal hypertension was calculated by the Youden Index for 11.2 kPa with an AUC of 0.78 (Fig. 6C).

**Fig. 7:**
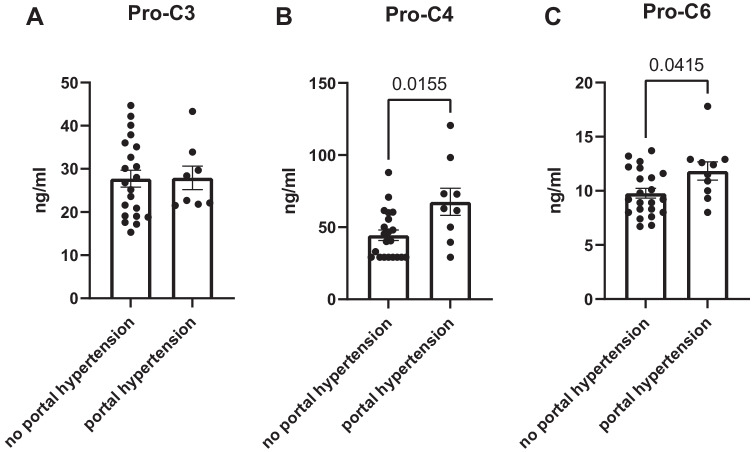
Serum collagen markers of liver fibrosis and fibrogenesis in patients with CVID. (**A**) Serum levels of Pro-C3 in CVID patients without portal hypertension (*n* = 21) compared to patients after diagnosis of portal hypertension (*n* = 8). (**B**) Serum levels of Pro-C4 in CVID patients without portal hypertension (*n* = 21) compared to patients after diagnosis of portal hypertension (*n* = 9). Statistical significance was assessed with Mann–Whitney test. Serum levels below the detection limit of the ELISA were set to 29.2 ng/ml as this is the lower limit of detection of the ELISA. (**C**) Serum levels of Pro-C6 in CVID patients without portal hypertension (*n* = 22) compared to patients after diagnosis of portal hypertension (*n* = 10). Statistical significance was assessed with Mann–Whitney test

Analysis of three serum derived biomarkers of liver collagen formation (Pro-C3, Pro-C4, and Pro-C6) [32–34] revealed significant increases in CVID patients with portal hypertension for Pro-C4 and for Pro-C6 (Fig. 7); however, none of these markers correlated with LSM values, diameter or Vmax of the portal vein, or crosswise or longitudinal spleen diameters (data not shown).

## Discussion

In this retrospective study, we characterize the clinical and sonographic appearance as well as blood laboratory values of CVID patients with portal hypertension. Our study shows that clinically significant portal hypertension occurs at a later time point in the disease course of CVID, when compared to other clinical manifestations such as granuloma, interstitial lung disease, lymphadenopathy, and splenomegaly. Patients that suffer from portal hypertension belong to a more severely affected patient collective with an increased mortality that co-displays multiple CVID organ manifestations compared to patients without portal hypertension. Of note, the patients with portal hypertension we identified in this study had a median diagnostic delay of 7 years (time between initial manifestation and diagnosis of CVID), compared to 4.4 years in the control group (*p* = 0.25) and 4.8–5 years described in the literature [35]. The cause, relevance, and consequences of this delay remain to be seen in larger studies.

In laboratory analysis, CVID patients with portal hypertension show elevated levels of ALT and γ-GT indicating cholestasis, whereas total bilirubin is not elevated in our cohort. Of note, many of the other laboratory parameters indicated a reduced synthesis capacity of the liver, such as lower albumin and Quick when compared to patients without manifest portal hypertension, although values were often still within the normal range. In line with this, only 2/21 biopsies revealed a cirrhosis, and most CVID patients suffer from a non-cirrhotic portal hypertension (NCPH). In sum, patients with CVID and nodular regenerative hyperplasia show a typical pathophysiological phenotype of non-cirrhotic portal hypertension that is characterized by a presinusoidal hepatic resistance due to obliteration of small and medium portal vein branches. These structural changes are not detectable by ultrasound. While there were significant differences between ultrasound measurements of patients with and without portal hypertension, most of them showed largely overlapping results and were of low discriminatory power. Surprisingly, the direction and the velocity of the portal venous flow were not altered, obfuscating the diagnosis of portal hypertension. The best marker in abdominal ultrasound of CVID patients with portal hypertension was the markedly increased spleen diameter compared to the non-portal hypertension CVID patient collective. As splenomegaly is a common phenomenon in more than 25% of patients with CVID [35] and may be caused not only by portal hypertension but also by lymphoproliferation, the presence of splenomegaly itself is highly sensitive, but not specific for portal hypertension. Only a particularly large spleen with a diameter above 16 cm was more strongly associated with portal hypertension and should prompt further evaluation in the affected patient.

As previously reported [20, 28], ultrasound-based transient elastography (FibroScan®) is of diagnostic value in CVID patients, in particular in those at risk for portal hypertension. LSM values above 20 kPa were regularly associated with relevant portal hypertension as suggested previously for non-CVID patients [36], but in our opinion already, all pathological LSM values > 6.5 kPa should prompt evaluation for secondary complications to reach a high sensitivity.

Among the previously suggested serum biomarkers of liver fibrosis and collagen formation (Pro-C3, Pro-C4, and Pro-C6) [32–34], only Pro-C4 and Pro-C6 but not Pro-C3 were elevated in CVID patients with portal hypertension. This may indicate that there is only a minor hepatic de novo synthesis of basement membrane (Pro-C4) and interstitial microfilaments (Pro-C6), but not of interstitial (Pro-C3) collagen in these patients, compatible with the absence of massive fibrotic extracellular matrix in the liver biopsies of most affected CVID patients. Accordingly, these parameters did not prove of additional value compared to sonographic determination of the spleen diameter.

In summary, the early detection of clinically significant portal hypertension in CVID patients remains a challenge. Especially patients with multiorgan disease, elevated γ-GT and a longitudinal spleen diameter greater than 16 cm need an evaluation for portal hypertension and secondary complications including liver elastography and gastroscopy and a close follow up by (semi)annual ultrasound as well as elastography every year to maximum every 2 years. We further suggest routine annual abdominal ultrasounds as useful to diagnose critical splenomegaly and other potential signs of portal hypertension in a timely fashion in all CVID patients. Of note, in our analysis, none of the blood parameters measured during routine follow up allowed for a good prediction of portal hypertension. In this study, the venous pressure gradients were not systematically measured in all patients, and diagnosis of portal hypertension was established clinically. While it was further not possible to identify the primary cause of liver disease in the majority of the patients with portal hypertension identified in this study, this will need to be a focus of future clinical studies on liver disease in CVID patients. There is still a large unmet need for novel biomarkers that allow early identification and monitoring of CVID patients with incipient and manifest portal hypertension so that pharmacological or endoscopic treatments to prevent complications arising from portal hypertension like potentially fatal upper gastrointestinal bleeding can be instituted.

## Supplementary Information

Below is the link to the electronic supplementary material.

Supplementary file1 IgG Substitution in relation to manifestation of portal hypertension in patients with CVID. Period and application route of immunoglobulin substitution in relationship to time of diagnosis of portal hypertension (red triangle) are depicted for all CVID patients with portal hypertension. (PDF 24.7 KB)

Supplementary file2 Laboratory parameters of CVID patients before and after diagnosis of portal hypertension. Laboratory values of patients with portal hypertension were compared before and after the onset of portal hypertension. A linear mixed model was computed to assess statistical significance. Blue line indicates the median. Grey shaded area reflects normal range. (PDF 883 KB)

## Data Availability

Not applicable.
