# Supplementary figures and images for: Evaluation of Laboratory and Sonographic Parameters for Detection of Portal Hypertension in Patients with Common Variable Immunodeficiency
Source: J Clin Immunol. 2022 Jul 11;42(8):1626–37. doi: 10.1007/s10875-022-01319-0 (PMC9700587; doi:10.1007/s10875-022-01319-0)

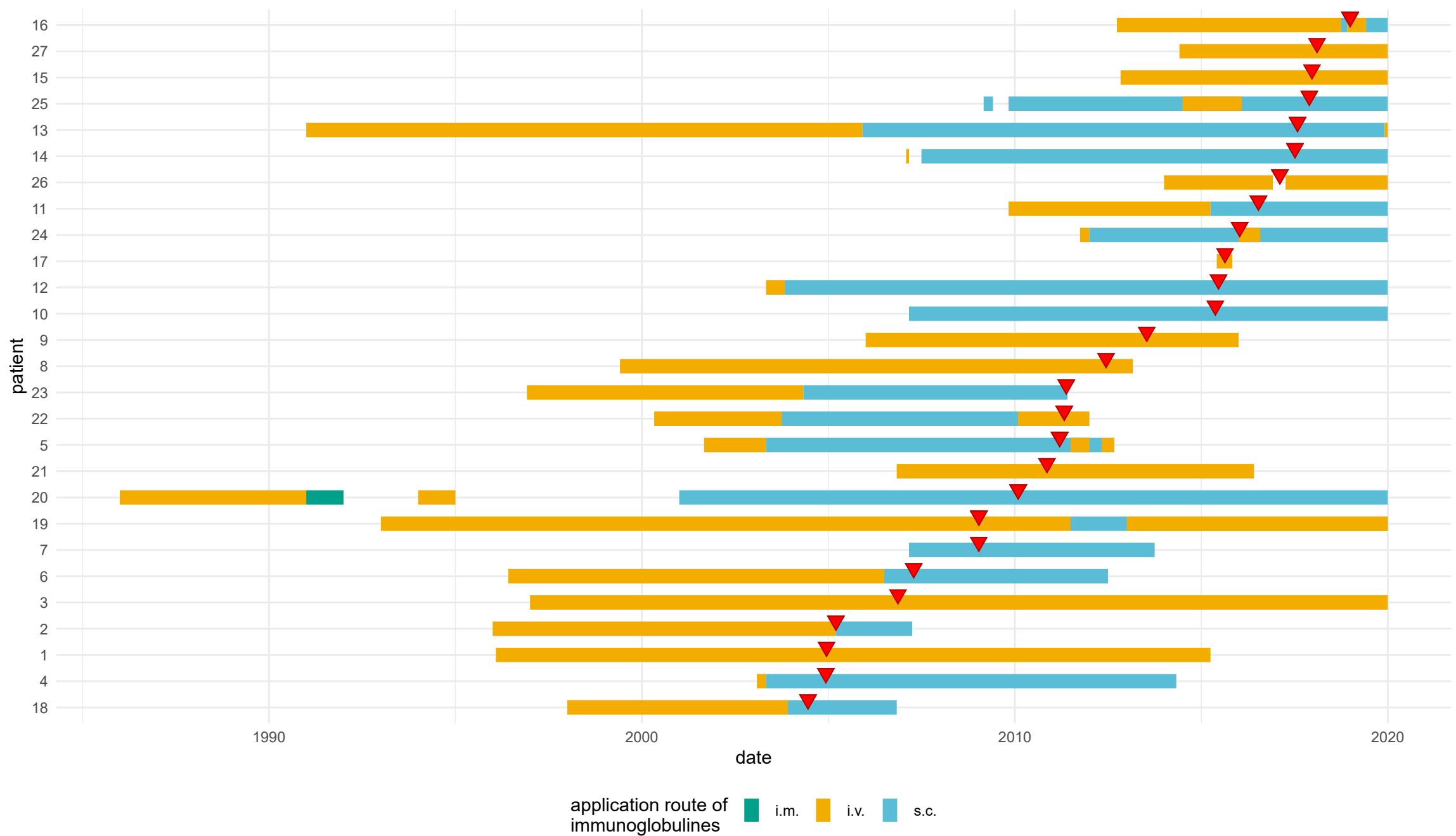

Supplement: Supplementary file 1 — Supplementary file1 IgG Substitution in relation to manifestation of portal hypertension in patients with CVID. Period and application route of immunoglobulin substitution in relationship to time of diagnosis of portal hypertension (red triangle) are depicted for all CVID patients with portal hypertension. (PDF 24.7 KB) [file 10875_2022_1319_MOESM1_ESM.pdf]
