# Supplementary material for: Evaluation of Laboratory and Sonographic Parameters for Detection of Portal Hypertension in Patients with Common Variable Immunodeficiency
Source: J Clin Immunol. 2022 Jul 11;42(8):1626–37. doi: 10.1007/s10875-022-01319-0 (PMC9700587; doi:10.1007/s10875-022-01319-0)
Supplement: Supplementary file 2 — Supplementary file2 Laboratory parameters of CVID patients before and after diagnosis of portal hypertension. Laboratory values of patients with portal hypertension were compared before and after the onset of portal hypertension. A linear mixed model was computed to assess statistical significance. Blue line indicates the median. Grey shaded area reflects normal range. (PDF 883 KB) [file 10875_2022_1319_MOESM2_ESM.pdf]

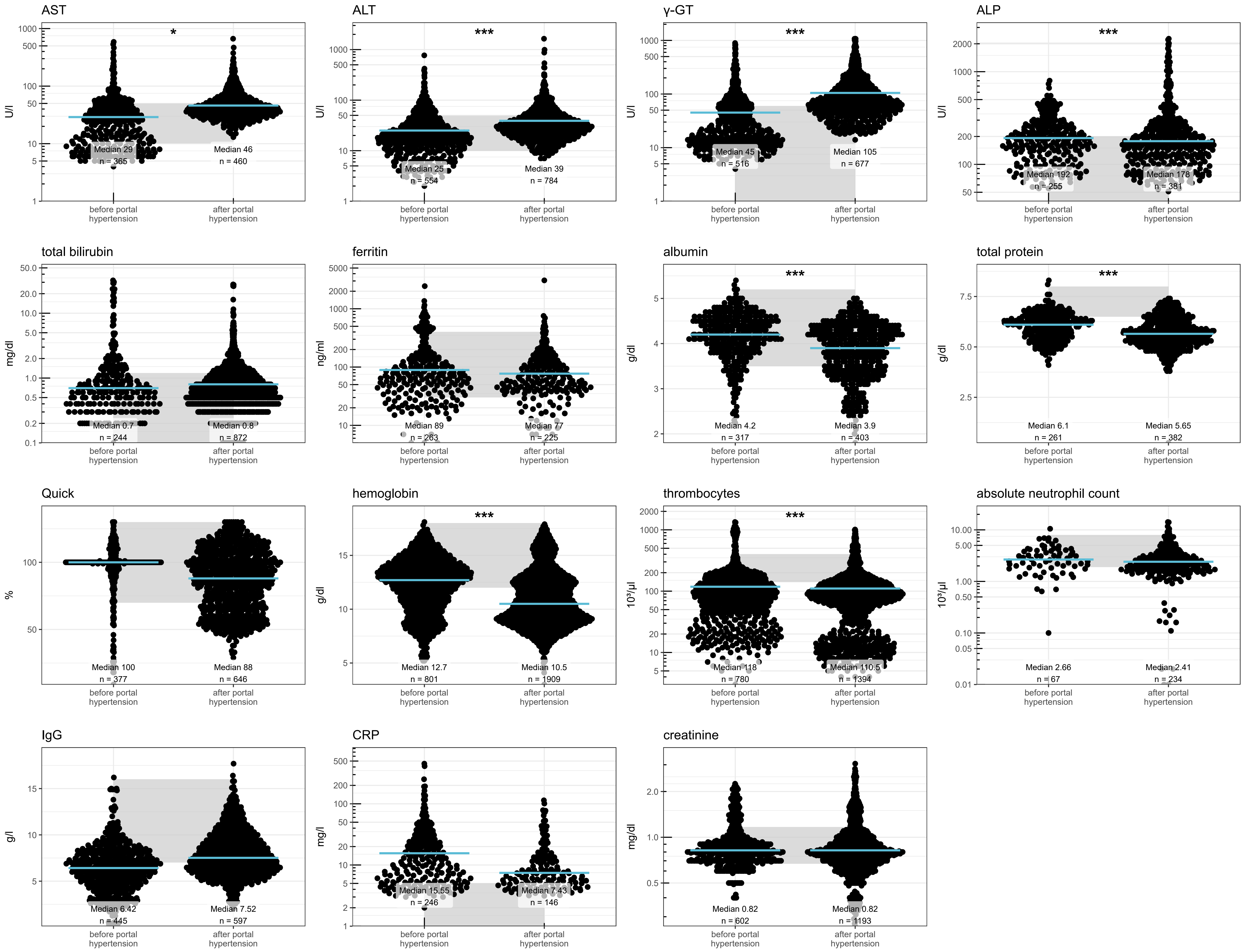
AST
*
U/l
Median 29
n = 365
Median 46
n = 460
ALT
***
U/l
Median 25
n = 554
Median 39
n = 784
γ-GT
***
U/l
Median 45
n = 516
Median 105
n = 677
ALP
***
U/l
Median 192
n = 255
Median 178
n = 381
total bilirubin
mg/dl
Median 0.7
n = 244
Median 0.8
n = 872
ferritin
ng/ml
Median 89
n = 263
Median 77
n = 225
albumin
***
g/dl
Median 4.2
n = 317
Median 3.9
n = 403
total protein
***
g/dl
Median 6.1
n = 261
Median 5.65
n = 382
Quick
%
Median 100
n = 377
Median 88
n = 646
hemoglobin
***
g/dl
Median 12.7
n = 801
Median 10.5
n = 1909
thrombocytes
***
10³/µl
Median 118
n = 780
Median 110.5
n = 1394
absolute neutrophil count
10³/µl
Median 2.66
n = 67
Median 2.41
n = 234
IgG
g/l
Median 6.42
n = 445
Median 7.52
n = 597
CRP
mg/l
Median 15.55
n = 246
Median 7.43
n = 146
creatinine
mg/dl
Median 0.82
n = 602
Median 0.82
n = 1193
before portal hypertension
after portal hypertension
